# Prognostic significance of RICTOR mutations in EGFR-mutant metastatic lung adenocarcinoma: a retrospective cohort study

**DOI:** 10.1007/s00428-026-04559-2

**Published:** 2026-05-04

**Authors:** Ali Aytac, Berkay Mehmet Ozata, Ibrahim Halil Erdogdu, Ali Alkan, Ozgur Tanriverdi

**Affiliations:** 1 Department of Medical Oncology, Mehmet Akif Inan Education and Research Hospital, Sanliurfa, Türkiye; 2 Department of Internal Medicine, Mugla Sitki Kocman University Faculty of Medicine, Mugla, Türkiye; 3 Department of Medical Pathology, Adnan Menderes University Faculty of Medicine, Aydin, Türkiye; 4 Department of Medical Oncology, Mugla Sitki Kocman University Faculty of Medicine, Mugla Universitesi Egitim ve Arastirma Hastanesi, Onkoloji Poliklinigi, Mugla, 48000 Türkiye

**Keywords:** Lung adenocarcinoma, EGFR mutation, RICTOR mutation, MTORC2, Co-mutation, Prognosis

## Abstract

RICTOR, a scaffold protein of the mTORC2 complex, regulates AKT signaling and has been implicated in tumor progression and therapy resistance across multiple cancers. However, the prognostic impact of RICTOR mutations in epidermal growth factor receptor (EGFR)-mutant lung adenocarcinoma remains unclear. This study aimed to evaluate the clinicopathological, molecular, and survival characteristics of patients with metastatic EGFR-mutant lung adenocarcinoma according to RICTOR mutation status. We retrospectively analyzed 235 patients diagnosed with de novo metastatic lung adenocarcinoma between 2018 and 2024 across three tertiary oncology centers. Patients with targetable oncogenic drivers other than EGFR (ALK, ROS1, HER2, KRAS G12C, BRAF V600E, RET, MET) were excluded. Next-generation sequencing (NGS) was performed using a hybrid-capture panel (Illumina TSO500). Clinical features, co-mutations, treatment responses, and overall survival (OS) were compared between RICTOR-mutant and wild-type subgroups within the EGFR-mutant cohort. Survival analyses employed Kaplan–Meier estimates, log-rank tests, and Cox regression modeling. Of the total cohort, 39 patients (17%) had EGFR-mutant tumors, of whom 15 (38%) carried RICTOR mutations. RICTOR-mutant cases were more likely to be former smokers and presented more frequently with bone and pleural metastases compared with wild type. Treatment patterns and RECIST v1.1 response rates did not significantly differ between groups. Median OS was significantly shorter in RICTOR-mutant versus RICTOR-wild patients (8 vs. 14 months, log-rank *p* < 0.001). In univariate analysis, RICTOR mutations were associated with inferior OS (HR 2.48, 95% CI 1.73–7.95, *p* = 0.03), and this association remained significant in multivariate analysis (HR 2.34, 95% CI 1.69–6.75, *p* = 0.021). Exploratory analyses suggested that RICTOR co-mutations with EGFR exon 19 deletions, exon 18 alterations, or exon 20 alterations were associated with poorer survival outcomes. RICTOR mutations were associated with a high-risk subset of EGFR-mutant metastatic lung adenocarcinoma characterized by more aggressive clinical features and worse survival. These findings suggest the need for prospective validation and support the potential integration of RICTOR status into molecular risk stratification frameworks for precision oncology.

**Supplementary Information:**

The online version contains supplementary material available at 10.1007/s00428-026-04559-2.

## Introduction

Lung cancer remains the leading cause of cancer-related mortality worldwide, accounting for approximately 1.8 million deaths annually and surpassing the combined mortality of breast, colorectal, and prostate cancers [1]. Non-small cell lung cancer (NSCLC) constitutes nearly 85% of cases, with adenocarcinoma being the most common histological subtype, particularly among never-smokers and younger patients [2]. Because of its indolent early course, lung adenocarcinoma is frequently diagnosed at advanced stages, where median overall survival (OS) with conventional chemotherapy historically ranged between 8 and 12 months [3].

The advent of molecular diagnostics has profoundly changed the management of advanced NSCLC. Among targetable oncogenic drivers, epidermal growth factor receptor (EGFR) mutations occur in 10–20% of Western and up to 50% of Asian patients with lung adenocarcinoma. The introduction of EGFR tyrosine kinase inhibitors (TKIs) has significantly improved survival compared with chemotherapy [4, 5]. Nevertheless, clinical outcomes among EGFR-mutant patients remain heterogeneous: while some individuals achieve durable responses with modern TKIs, others experience early progression despite optimal therapy. This variability underscores the importance of understanding the molecular context of EGFR-driven tumors, particularly the role of co-mutations and interactions with parallel signaling pathways [6–9]. In this context, alterations affecting downstream signaling networks have been increasingly recognized as potential modulators of treatment response and resistance to EGFR-targeted therapies.

One pathway of particular relevance is the PI3K–AKT–mTOR signaling cascade, which is involved in tumor proliferation, survival, and therapeutic resistance. Within this pathway, the mechanistic target of rapamycin complex 2 (mTORC2), scaffolded by RICTOR, regulates AKT phosphorylation at Ser473 and influences cellular growth, metabolism, and cytoskeletal dynamics [10–12]. Activation of the mTORC2–AKT axis has been implicated in oncogenic signaling independent of upstream receptor tyrosine kinases and may contribute to bypass mechanisms that attenuate the efficacy of EGFR inhibition. Preclinical and clinical evidence suggests that dysregulation of this pathway can promote tumor progression, enhance metastatic potential, and reduce sensitivity to targeted therapies, including EGFR-TKIs [11, 12].

Emerging evidence may further suggests that RICTOR alterations, including amplification and overexpression, may define a biologically aggressive subset of tumors and have been associated with resistance to targeted treatments in multiple cancer types, including lung cancer [13, 14]. These observations raise the possibility that RICTOR-mediated signaling may interact with EGFR-driven oncogenesis and influence clinical outcomes. However, while RICTOR amplification has been relatively well characterized, the clinical and prognostic significance of RICTOR mutations—as distinct genomic events—remains poorly defined, particularly in the setting of EGFR-mutant lung adenocarcinoma [15, 16].

To address this gap, we investigated the clinical, pathological, and molecular characteristics of patients with EGFR-mutant metastatic lung adenocarcinoma according to RICTOR mutation status and evaluated their prognostic implications in a real-world cohort.

## Methods

### Study design and patient selection

This retrospective multi-center cohort study included 235 consecutive patients diagnosed with *de novo* metastatic lung adenocarcinoma between January 2018 and December 2024 across three tertiary oncology centers. Patients with recurrent disease after prior early-stage treatment were excluded to ensure a strictly de novo cohort. The study was approved by the Institutional Ethics Committee (Medical Sciences Ethics Committee of Mugla Sitki Kocman University Approval Number: 240038, Date: September 11, 2024) and conducted in accordance with the Declaration of Helsinki.

Patients were identified from institutional databases of the participating centers. Eligible patients had histopathologically confirmed adenocarcinoma according to the 2021 WHO classification, with radiologically or histologically verified distant metastases at diagnosis [17]. Diagnostic material was obtained primarily via core needle or endobronchial biopsies, supplemented by cytology when necessary. Immunohistochemistry (IHC) for thyroid transcription factor-1 (TTF-1) and Napsin A supported diagnosis in equivocal cases [18].

Inclusion criteria were: (1) availability of next-generation sequencing (NGS) results and (2) complete clinical data (demographics, treatment history, follow-up, and survival status). Patients with targetable oncogenic drivers other than EGFR—including anaplastic lymphoma kinase (ALK), c-ros oncogene 1 (ROS1), human epidermal growth factor receptor 2 (HER2), Kirsten rat sarcoma viral oncogene homolog G12C (KRAS G12C), v-raf murine sarcoma viral oncogene homolog B V600E (BRAF V600E), rearranged during transfection (RET), and mesenchymal–epithelial transition factor exon 14 skipping (MET)—were excluded, as they would typically receive alternative targeted therapies. Additional exclusion criteria included history of prior or concurrent malignancy, inadequate tissue for molecular testing, or incomplete follow-up.

Clinical data were retrospectively extracted from the electronic medical record systems of the participating centers. Demographic, clinical, treatment, and survival data were cross-checked by two independent investigators to improve data accuracy. Clinical and molecular datasets were integrated using unique patient identifiers.

### Molecular testing and mutation analysis

Genomic profiling was performed on formalin-fixed paraffin-embedded (FFPE) tumor samples using a validated hybrid-capture NGS panel (Illumina TruSight Oncology 500, TSO500). The panel interrogates > 500 cancer-related genes and detects single nucleotide variants (SNVs), insertions/deletions (indels), copy-number alterations, and selected fusions. Median sequencing coverage exceeded 500×, with a minimum depth threshold of 200× and a variant allele frequency (VAF) cut-off of 5% for calling. Variant interpretation followed ACMG/AMP guidelines, and only pathogenic or likely pathogenic variants were included in analyses.

For each patient, only genes reported as pathogenic or likely pathogenic in the NGS report were extracted and included in the analysis. Variants of uncertain significance (VUS) and benign polymorphisms were excluded. In addition to EGFR and RICTOR, clinically relevant alterations in genes such as KRAS, TP53, PIK3CA, ALK, ROS1, BRAF, and others were evaluated as part of the comprehensive panel.

#### EGFR testing

Variants in exons 18–21 were systematically recorded, including common sensitizing mutations (exon 19 deletions, L858R in exon 21), atypical mutations (G719X, L861Q, S768I), and exon 20 insertions. Resistance-associated variants (T790M, C797S) were also documented when present at baseline, although none were detected in this cohort [19].

#### RICTOR testing

RICTOR alterations (missense variants, in-frame deletions, nonsense or frameshift mutations) were identified and validated by NGS. Copy-number alterations were excluded from the primary analysis to focus on mutational events. Variant classification was supported by curated databases, including COSMIC, ClinVar, and OncoKB, to improve consistency and clinical relevance [20].

Co-occurring mutations in KRAS (non-G12C) and TP53 were also extracted for comparative and survival analyses.

### Clinicopathological data

Collected variables included demographic factors (age, sex, smoking history), clinical features (ECOG performance status, comorbidities including cardiovascular disease, diabetes mellitus, and chronic pulmonary disorders), metastatic sites at presentation, histopathological features (tumor grade, TTF-1 expression), and PD-L1 status.

PD-L1 expression was assessed on FFPE sections using the Ventana SP263 antibody and scored as tumor proportion score (TPS): <1%, 1–49%, or ≥ 50% [21].

### Treatment data

First-line therapies were categorized as platinum-based chemotherapy, EGFR tyrosine kinase inhibitors (TKIs), or immune checkpoint inhibitors (ICIs). All patients in the EGFR-mutant subgroup uniformly received EGFR-TKIs (osimertinib, erlotinib, gefitinib). Radiological tumor response was evaluated every 8–12 weeks using computed tomography (CT) scans according to the Response Evaluation Criteria in Solid Tumors (RECIST) version 1.1, and best overall response (complete response, partial response, stable disease, or progressive disease) was recorded. Second-line treatment modalities (chemotherapy, subsequent TKI, or ICI) were also documented.

### Statistical analysis

Continuous variables were assessed for normality using the Kolmogorov–Smirnov test. Non-normally distributed data were summarized as medians with interquartile range (IQR), and categorical variables were reported as counts and percentages. Between-group comparisons were performed using Pearson’s chi-square or Fisher’s exact test (categorical variables) and the Mann–Whitney U test (continuous variables).

Overall survival (OS) was defined as the time from diagnosis to death or last follow-up. Median follow-up duration for the entire cohort was 16.2 months (IQR, 11.5–21.4), estimated using the reverse Kaplan–Meier method. Kaplan–Meier survival curves were generated, and differences between groups were assessed with the log-rank test. PFS could not be reliably assessed due to incomplete and heterogeneous radiologic follow-up data in this retrospective cohort; therefore, overall survival was chosen as the primary endpoint.

Univariate Cox regression analyses were conducted to evaluate associations of clinical and molecular variables with OS. Variables with *p* < 0.05 in univariate testing were entered into multivariate Cox regression models using backward stepwise elimination. Collinearity was checked before model inclusion. Hazard ratios (HRs) and 95% confidence intervals (CIs) were reported. Proportional hazards assumptions were verified using Schoenfeld residuals.

Finally, exploratory univariate analyses were performed to assess the prognostic effect of co-occurring RICTOR mutations with specific EGFR variants (e.g., exon 19 deletions, exon 20 insertions, uncommon exon 18 alterations). These subgroup analyses were descriptive and hypothesis-generating, not included in multivariate models, and results were interpreted cautiously due to small sample sizes.

All statistical tests were two-sided, with *p* < 0.05 considered significant. Analyses were performed using IBM SPSS Statistics version 25.0.

## Results

### Baseline characteristics

A total of 235 patients with lung adenocarcinoma were included in the study, of whom 39 (17%) harbored an EGFR mutation and 196 (83%) were EGFR wild type (Table 1).

**Table 1:** Baseline Demographic, Clinical, and Histopathological Characteristics of Patients Stratified by EGFR Mutation Status

Characteristic	All Patients (*n* = 235)	EGFR mutant (*n* = 39)	EGFR wild (*n* = 196)	*p*-value*
Gender, *n* (%)				
Male	137 (58)	15 (39)	122 (62)	**0.048**
Female	98 (42)	24 (61)	74 (38)	
Age, *n* (%)				
<65 years	96 (41)	26 (67)	70 (36)	**0.042**
≥65 years	139 (59)	13 (33)	126 (64)	
Smoking History, n (%)				
Current smoker	94 (40)	6 (15)	88 (45)	**0.044**
Former smoker	104 (44)	12 (31)	92 (47)	
Never smoked	37 (16)	21 (54)	16 (8)	
ECOG Performance Status, n (%)				
1	174 (74)	25 (64)	149 (76)	0.374
2	61 (26)	14 (36)	47 (24)	
Comorbidity, n (%)				
Absent	106 (45)	19 (49)	87 (44)	0.317
Present	129 (55)	20 (51)	109 (56)	
Systemic Diseases, n (%)				
None	106 (45)	19 (49)	87 (44)	0.096
COPD	112 (46)	11 (28)	101 (51)	
Hypertension	69 (29)	17 (43)	52 (26)	
Diabetes Mellitus	48 (20)	9 (25)	39 (20)	
Ischemic Heart Disease	33 (14)	6 (15)	27 (14)	
Hypothyroidism	9 (4)	1 (2)	8 (4)	
Metastasis at Diagnosis, n (%)				
Lung	116 (49)	22 (56)	94 (48)	0.069
Liver	61 (26)	9 (23)	52 (26)	
Bone	58 (25)	9 (23)	49 (25)	
Adrenal gland	48 (20)	4 (10)	44 (22)	
Pleural effusion	24 (10)	7 (18)	17 (9)	
Brain	19 (8)	2 (5)	17 (9)	
TTF-1 Expression, n (%)				
Positive	184 (78)	33 (85)	151 (77)	0.412
Negative	51 (22)	6 (15)	45 (23)	
Tumor Grade, n (%)				
Grade 1	24 (10)	2 (5)	22 (11)	0.245
Grade 2	174 (74)	35 (90)	139 (71)	
Grade 3	37 (16)	2 (5)	35 (18)	
PD-L1 Expression, n (%)				
Negative (< 1%)	129 (55)	31 (80)	98 (50)	0.074
1–49%	69 (29)	6 (15)	63 (32)	
≥50%	37 (16)	2 (5)	35 (18)	
RICTOR mutation				
Present	37 (16)	15 (39)	22 (11)	0.066
Absent	198 (84)	24 (61)	174 (89)	
TP53 mutation				
Present	34 (15)	14 (36)	20 (10)	**0.044**
Absent	201 (85)	25 (64)	176 (90)	
KRAS mutation (except for G12C)				
Present	33 (14)	11 (28)	22 (11)	0.072
Absent	202 (86)	28 (72)	174 (89)	

*EGFR* Epidermal Growth Factor Receptor, *ECOG* Eastern Cooperative Oncology Group, *COPD* Chronic Obstructive Pulmonary Disease, *TTF-1* Thyroid Transcription Factor 1, *PD-L1* Programmed Death Ligand 1,*KRAS* Kirsten Rat Sarcoma Virus, *BRAF* B-Raf Proto-Oncogene

Statistical Note: *P*-values are presented as unadjusted unless otherwise specified. Bonferroni correction was applied only for selected multiple comparisons. Comparisons between categorical variables were performed using chi-square or Fisher’s exact test where appropriate

Patients with EGFR mutations were more frequently female (61% vs. 38%, *p* = 0.048), younger than 65 years (67% vs. 36%, *p* = 0.042), and never-smokers (54% vs. 8%, *p* = 0.044) compared with EGFR wild-type patients. No significant differences were observed regarding ECOG performance status, comorbidities, systemic diseases, metastatic sites, TTF-1 expression, tumor grade, or PD-L1 expression. PD-L1 distribution was broadly similar between EGFR-mutant and wild-type cases (< 1%: 36% vs. 39%; 1–49%: 41% vs. 38%; ≥50%: 23% vs. 23%).

With respect to genomic alterations, EGFR-mutant patients were more likely to harbor concomitant TP53 mutations (36% vs. 10%, *p* = 0.044) and tended to have more frequent RICTOR mutations (39% vs. 11%, *p* = 0.066), although the latter did not reach statistical significance after Bonferroni correction. By contrast, KRAS mutations (non-G12C) were more common in EGFR-mutant compared with wild-type tumors (28% vs. 11%, *p* = 0.072).

Detailed annotation of RICTOR variants, including cDNA changes, protein alterations, variant allele frequency, and pathogenicity classification, is provided in Supplementary Table S1.

### Characteristics of EGFR-mutant patients according to RICTOR status

Within the EGFR-mutant subgroup (*n* = 39), 15 patients carried RICTOR mutations and 24 were RICTOR wild type (Table 2). Demographic characteristics, ECOG performance status, and comorbidity profiles were comparable between groups.

**Table 2:** Demographic, Clinical, Histopathological, and Molecular Characteristics of EGFR-Mutant Patients Stratified by RICTOR Mutation Status

Characteristic	All Patients (*n* = 39)	RICTOR Mutant (*n* = 15)	RICTOR Wild (*n* = 24)	*p*-value
Demographic and Clinical Features				
Gender, *n* (%)				
Male	15 (39)	6 (40)	9 (38)	0.542
Female	24 (61)	9 (60)	15 (62)	
Age, n (%)				
<65 years	26 (67)	9 (60)	17 (71)	0.337
≥65 years	13 (33)	6 (40)	7 (29)	
Smoking History, n (%)				
Current smoker	6 (15)	1 (7)	5 (21)	0.037
Former smoker	12 (31)	9 (60)	3 (12)	
Never smoked	21 (54)	5 (33)	16 (67)	
ECOG Performance Status, n (%)				
1	25 (64)	11 (73)	14 (58)	0.212
2	14 (36)	4 (27)	10 (42)	
Comorbidity, n (%)				
Absent	19 (49)	9 (60)	10 (42)	0.252
Present	20 (51)	6 (40)	14 (58)	
Systemic Diseases, n (%)				
None	19 (49)	9 (60)	10 (42)	0.112
COPD	11 (28)	7 (47)	16 (67)	
Hypertension	17 (43)	4 (27)	13 (54)	
Diabetes Mellitus	9 (25)	1 (7)	8 (33)	
Ischemic Heart Disease	6 (15)	1 (7)	5 (21)	
Hypothyroidism	1 (2)	0	1 (4)	
Metastasis at Diagnosis, n (%)				
Lung	22 (56)	11(73)	11 (46)	**0.037**
Liver	9 (25)	4 (27)	5 (21)	
Bone	9 (25)	6 (40)	3 (12)	
Adrenal gland	4 (10)	2 (13)	2 (8)	
Pleural effusion	7 (18)	5 (33)	2 (8)	
Brain	2 (5)	0	2 (8)	
TTF-1 Expression, n (%)				
Positive	33 (85)	13 (87)	20 (83)	0.416
Negative	6 (15)	2 (13)	4 (17)	
Tumor Grade, n (%)				
Grade 1	2 (5)	1(7)	2 (8)	0.676
Grade 2	35 (90)	13 (87)	20 (83)	
Grade 3	2 (5)	1(7)	2 (8)	
PD-L1 Expression, n (%)				
Negative (< 1%)	31 (80)	11 (73)	20 (83)	0.317
1–49%	6 (15)	3 (20)	3 (12)	
≥50%	2 (5)	1 (7)	1 (4)	
TP53 mutation				
Present	14 (36)	6 (40)	6 (25)	0.094
Absent	25 (64)	9 (60)	18 (75)	
KRAS mutation (except for G12C)				
Present	11 (28)	5 (33)	6 (25)	0.194
Absent	28 (72)	10 (67)	18 (75)	
1 st line treatment				
Osimertinib	17	7 (47)	10 (42)	0.379
Erlotinib	14	5 (33)	9 (37)	
Gefitinib	8	3 (20)	5 (21)	
Treatment Response				
Complete response	5	1 (7)	4 (17)	0.294
Partial response	27	11 (73)	16 (67)	
Stable disease	5	2 (13)	3 (12)	
Progression	2	1 (7)	1 (4)	
2nd line treatment				
Chemotherapy	11	5 (33)	6 (25)	0.216
Targeted TKIs therapy	19	7 (47)	12 (50)	
ICI monotherapy	9	3 (20)	6 (25)	

*ECOG* Eastern Cooperative Oncology Group, *COPD* Chronic Obstructive Pulmonary Disease, *TTF-1* Thyroid Transcription Factor 1, *PD-L1* Programmed Death Ligand 1,*KRAS* Kirsten Rat Sarcoma Virus, *BRAF* B-Raf Proto-Oncogene, *TKIs* Tyrosine Kinase Inhibitors

Statistical Note: P-values are presented as unadjusted unless otherwise specified. Bonferroni correction was applied only for selected multiple comparisons. Chi-square or Fisher’s exact test was applied for categorical comparisons. The table highlights baseline differences in clinical features, metastasis patterns, treatment modalities, and outcomes between RICTOR-mutant and RICTOR-wild patients

Smoking history, however, differed significantly: RICTOR-mutant patients were more likely to be former smokers (60% vs. 12%), whereas never-smoking status predominated in the RICTOR-wild group (67%, *p* = 0.037). At diagnosis, RICTOR-mutant patients more frequently presented with bone and pleural metastases (40% vs. 12% and 33% vs. 8%, respectively; *p* = 0.037).

Histopathological features, including TTF-1 expression (87% vs. 83%), tumor grade, and PD-L1 status, did not differ significantly between the two groups. First-line EGFR-TKI treatment patterns were balanced (osimertinib, erlotinib, gefitinib), and objective response rates were similar (79% vs. 83%). The distribution of stable disease (13% vs. 13%) and progressive disease (8% vs. 4%) was likewise comparable.

### Spectrum of EGFR mutations

The distribution of EGFR variants is summarized in Table 3. Exon 19 deletions were the most frequent alteration, with classical variants such as p.E746_A750del accounting for 27% of RICTOR-mutant and 42% of RICTOR-wild tumors. The exon 21 L858R substitution was the second most common variant, detected in 20% and 25% of RICTOR-mutant and wild-type patients, respectively.

**Table 3:** Distribution of EGFR Mutation Subtypes in the Current Study Compared with Literature and Stratified by RICTOR Mutation Status

Exon	Variant	HGVS c.	Frequency in Literature	Current Study (*n*, %)	
				mRICTOR (*n* = 15)	wRICTOR (*n* = 24)
Exon 19	**p.E746_A750del(1)**	**c.2235_2249del15**	**45.0%**	4 (27)	10 (42)
	**p.E746_A750del(2)**	**c.2236_2250del15**	**19.6%**	2 (13)	3 (12)
	p.L747_P753 > S	complex delins	8.4%	0	0
	p.L747_T751del	varies; e.g., c.2240_2252del	4.3%	0	0
	p.L747_A750 > P	c.2239_2248delinsCC (example)	3.4%	1 (7)	0
	p.E746_S752 > V(2)	complex delins	3.2%	0	0
	p.E746_S752 > V	complex delins	1.6%	0	0
	p.L747_S752del	varies; e.g., c.2239_2254del	1.4%	0	0
	p.E746_A750del(3)	c.2236_2249del14	0.7%	0	0
	p.E746_P753 > MS	c.2236_2257 > atgt/> atgtc	0.4% (two single-case entries)	0	0
	p.E746_T751 > KV	c.2235_2252 > ggt	0.2% (single case)	1 (7)	0
	p.E746_A750 > HS	c.2236_2248 > ctaa	0.2% (single case)	0	0
	p.E746_T750del(4)	c.2236_2251 > a	0.2% (single case)	0	1 (4)
	p.E746_T751 > FPT	c.2236_2251 > tttccaa	0.2% (single case)	0	0
	p.E746_T751 > L	c.2236_2252 > ct	0.2% (single case)	1 (7)	0
	p.E746_S752 > IP	c.2236_2255 > atacc	0.2% (single case)	0	0
Exon 21	**p.L858R**	**c.2573T > G**	**≈ 35–45% of EGFR-mutant LUAD**	3 (20)	6 (25)
	p.L861Q	c.2582T > A	≈ 2–4% of EGFR-mutant LUAD	1 (7)	2 (8)
Exon 18	p.G719A	c.2156G > C	≈ 1% of EGFR-mutant LUAD	0	0
	p.G719C	c.2155G > T	≈ 1%	1 (7)	0
	p.G719S	c.2155G > A	≈ 1%	0	1 (4)
	p.E709K	c.2125G > A	< 0.5%	0	0
	p.E709_T710delinsD	c.2127_2131delinsGAT	single cases	0	0
Exon 20	p.S768I	c.2303G > T	≈ 1–2% of EGFR-mutant LUAD	1 (7)	1 (4)
	Exon 20 insertions (multiple subtypes)	various (c.2290_2291ins… etc.)	≈ 5–10% of EGFR-mutant LUAD	0	0
	p.T790M	c.2369 C > T	≈ 1% de novo	0	0

*EGFR* Epidermal Growth Factor Receptor, *LUAD* = Lung Adenocarcinoma, *HGVS* Human Genome Variation Society

Statistical Note: Frequencies are expressed as absolute numbers and percentages. The table summarizes the spectrum of EGFR variants observed in the current cohort (n=39) and compares them with reported frequencies in the literature, further stratified by RICTOR mutation status

Less frequent alterations included L861Q substitutions (7–8%), exon 18 G719 variants (4–7%), and the exon 20 S768I substitution (4–7%). Rare exon 19 complex indels (e.g., p.L747_A750 > P, p.E746_T751 > L) were observed only in the RICTOR-mutant subgroup, while an uncommon exon 20 deletion (p.E746_T750del) appeared exclusively in the RICTOR-wild group. Importantly, no *de novo* T790M or exon 20 insertion variants were identified in this cohort.

### Overall survival analyses

**Fig. 1:**
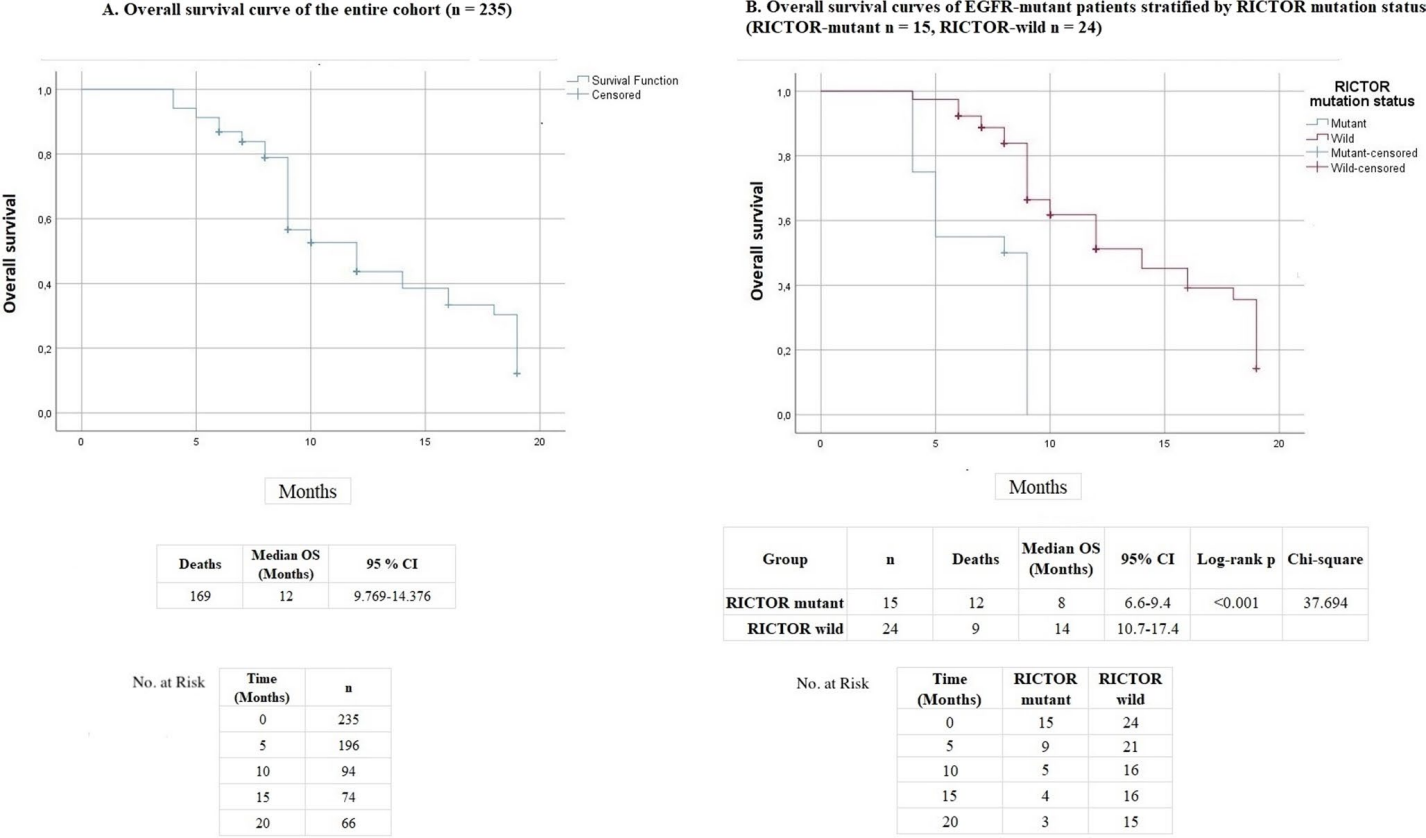
Overall survival analyses. (**A**) Overall survival curve of the entire cohort (n = 235). (**B**) Overall survival curves of EGFR-mutant patients stratified by RICTOR mutation status (RICTOR-mutant n = 15, RICTOR-wild n = 24)

The median OS for the entire cohort (*n* = 235) was 12 months (95% CI, 9.8–14.4), with 169 deaths observed during follow-up (Fig. 1 A). The median follow-up duration was 16.2 months (IQR, 11.5–21.4), estimated using the reverse Kaplan–Meier method (Fig. 1 A). The estimated 12- and 24-month OS rates for the whole cohort were 50% and 26%, respectively.

Within the EGFR-mutant subgroup, RICTOR status stratified outcomes. Patients harboring RICTOR mutations (*n* = 15) had a median OS of 8 months (95% CI, 6.6–9.4), significantly shorter than the 14 months (95% CI, 10.7–17.4) observed in RICTOR-wild patients (*n* = 24) (log-rank *p* < 0.001, χ² = 37.694) (Fig. 1B).

### Prognostic impact of clinical and genomic factors

Univariate Cox regression analyses identified liver metastasis (HR 1.69, 95% CI 1.35–4.15, *p* = 0.04), KRAS mutations (HR 2.16, 95% CI 1.35–4.24, *p* = 0.03), TP53 mutations (HR 2.61, 95% CI 1.39–5.19, *p* = 0.04), and RICTOR mutations (HR 2.48, 95% CI 1.73–7.95, *p* = 0.03) as factors associated with inferior OS. In multivariate analysis, only RICTOR mutations remained independently associated with worse survival (HR 2.34, 95% CI 1.69–6.75, *p* = 0.021), whereas liver metastasis, KRAS, and TP53 lost significance (Table 4).

**Table 4:** Prognostic Impact of Clinical and Genomic Factors on Overall Survival (Cox Regression Analysis)

Variable	Univariate			Multivariate		
	HR	95% CI	*p*-value	HR	95% CI	*p*-value
Metastatic site (Liver vs. Others)	**1.69**	**1.349–4.149**	**0.04**	1.44	0.717–3.569	0.383
KRAS Mutation (Mutant vs. Wild)	**2.16**	**1.349–4.241**	**0.03**	1.96	0.794–4.324	0.426
TP53 Mutation (Mutant vs. Wild)	**2.61**	**1.385–5.194**	**0.04**	2.06	0.676–3.216	0.212
RICTOR Mutation (Mutant vs. Wild)	**2.48**	**1.734–7.948**	**0.03**	**2.34**	**1.694–6.749**	**0.021**

*HR* Hazard Ratio, *CI* Confidence Interval, *TTF-1* Thyroid Transcription Factor 1, *EGFR* Epidermal Growth Factor Receptor, *KRAS* Kirsten Rat Sarcoma Virus, *TP53* Tumor Protein p53, *RICTOR* Rapamycin-Insensitive Companion of mTOR

Statistical Note: Both univariate and multivariate Cox regression analyses were performed. Variables with p < 0.05 in univariate analysis were included in multivariate analysis using backward stepwise elimination after testing for multicollinearity (via variance inflation factors)

### Exploratory analysis of RICTOR co-mutations

Exploratory univariate analyses assessed the impact of RICTOR co-occurrence with specific EGFR variants (Table 5). Patients harboring both RICTOR and EGFR mutations exhibited significantly shorter survival compared with those without this combination (HR 2.74, 95% CI 1.42–4.80, *p* < 0.001).

**Table 5:** Exploratory Univariate Analysis of RICTOR Co-Mutations and Their Association with Overall Survival

Variable	Univariate		
	HR	95% CI	*p*-value
Co-occurrence of RICTOR Mutation and EGFR Mutation (Present vs. Absent)	**2.74**	**1.419–4.796**	**< 0.001**
Co-occurrence of RICTOR Mutation and EGFR Exon 19 Mutation (Present vs. Absent)	**2.49**	**1.334–6.948**	**0.033**
Co-occurrence of RICTOR Mutation and Uncommon EGFR Exon 19 Mutation (Present vs. Absent)	**2.94**	**1.494–4.249**	**< 0.001**
Co-occurrence of RICTOR Mutation and EGFR Exon 21 Mutation (Present vs. Absent)	1.48	0.449–5.246	0.374
Co-occurrence of RICTOR Mutation and Uncommon EGFR Exon 21 Mutation (Present vs. Absent)	1.69	0.581–4.094	0.394
Co-occurrence of RICTOR Mutation and EGFR Exon 18 Mutation (Present vs. Absent)	**2.83**	**1.489–7.437**	**0.048**
Co-occurrence of RICTOR Mutation and EGFR Exon 20 Mutation (Present vs. Absent)	**2.79**	**1.589–6.437**	**0.037**

*HR* Hazard Ratio, *CI* Confidence Interval, *EGFR* Epidermal Growth Factor Receptor, *RICTOR* Rapamycin-Insensitive Companion of mTOR

Statistical Note: Univariate Cox regression was used to assess associations between RICTOR mutation co-occurrence with specific EGFR mutation subtypes and overall survival. Multivariate analysis was not performed due to overlapping cases and limited sample size

In these exploratory analyses, the most unfavorable survival outcomes were observed in cases where RICTOR mutations coexisted with EGFR exon 19 deletions (HR 2.49, 95% CI 1.33–6.95, *p* = 0.033), uncommon exon 19 variants (HR 2.94, 95% CI 1.49–4.25, *p* < 0.001), exon 18 mutations (HR 2.83, 95% CI 1.49–7.44, *p* = 0.048), or exon 20 alterations (HR 2.79, 95% CI 1.59–6.44, *p* = 0.037). By contrast, no significant association with survival was observed for exon 21 mutations. Given the small sample sizes, these findings should be considered hypothesis-generating and interpreted cautiously.

## Discussion

The management of EGFR-mutant metastatic lung adenocarcinoma has been transformed by EGFR tyrosine kinase inhibitors (TKIs), which achieve substantially longer survival than chemotherapy. Across pivotal trials, median overall survival (OS) typically exceeds 18 months and may approach or surpass three years with contemporary agents. In the FLAURA trial, first-line osimertinib achieved a median OS of 38.6 months, establishing a new benchmark for this population [22, 23]. Dacomitinib also improved survival versus gefitinib in ARCHER 1050 (median OS 34.1 months) [24], and afatinib demonstrated prolonged survival in LUX-Lung 3 among patients with sensitizing EGFR mutations [25]. In later-line settings, AURA3 and real-world cohorts reported OS in the 18–20-month range among TKI-treated patients [26, 27]. Collectively, these data support the expectation that most patients with sensitizing EGFR mutations have comparatively favorable outcomes under targeted therapy.

Against this backdrop, our findings reveal a marked deviation for the subgroup harboring RICTOR mutations. Within the EGFR-mutant cohort, RICTOR-mutant cases experienced a median OS of only 8 months—considerably shorter than both historical trial benchmarks and the RICTOR-wild subgroup in our study—suggesting that RICTOR mutation status may be associated with inferior outcomes under EGFR-targeted therapy.

The biological plausibility of this observation is strong. RICTOR scaffolds the mTORC2 complex, which phosphorylates AKT at Ser473 and sustains downstream pro-survival signaling [28–31]. Preclinical data indicate that hyperactive mTORC2 can blunt the efficacy of upstream receptor blockade by maintaining PI3K–AKT signaling, thereby promoting TKI resistance [28–31]. Moreover, RICTOR genomic alterations—most extensively studied as amplifications—have been associated with aggressive disease and potential sensitivity to dual mTORC1/2 inhibition in subsets of lung cancers [34, 35]. According to the literature knowledge, persistent activation of the mTORC2–AKT axis may serve as a bypass signaling mechanism, allowing tumor cells to maintain survival and proliferative signaling despite effective EGFR inhibition. This mechanism has been proposed as a contributor to both primary and adaptive resistance to EGFR-TKIs, particularly in tumors with co-activation of parallel survival pathways [35]. Our clinical signal of poor OS in RICTOR-mutant EGFR-driven tumors is consistent with this mechanistic framework of incomplete pathway suppression under EGFR-targeted therapy.

The co-mutational context further supports a high-risk biology. We observed frequent co-occurrence of RICTOR with alterations in TP53 and KRAS, genomic events that have been independently linked to adverse outcomes in NSCLC and in EGFR-mutant disease specifically [32, 33]. These patterns are consistent with the broader view that composite genomic landscapes, rather than single drivers, shape prognosis and modulate therapeutic vulnerability [32, 33, 35].

Clinically, three implications arise. First, not all EGFR-mutant tumors are biologically equivalent: canonical exon 19 deletions and L858R generally predict benefit from TKIs, but concomitant RICTOR mutations may identify a subgroup with worse survival in our cohort. Second, these data raise the hypothesis that dual-pathway strategies—co-targeting EGFR and mTORC2/AKT—may be required to overcome resistance in this subset; preclinical studies report synergistic anti-tumor effects with combined blockade of mTORC2-related signaling [34, 35]. Third, given the relatively high prevalence of RICTOR alterations in our EGFR-mutant cohort, systematic RICTOR assessment within clinical molecular profiling may help refine risk stratification and inform treatment prioritization.

The observed frequency of RICTOR mutations in our EGFR-mutant cohort appears higher than that reported in large genomic datasets, where prevalence is generally around 3–4%. Several factors may account for this discrepancy. First, our analysis was restricted to a de novo metastatic population enriched for EGFR-mutant tumors, a biologically and clinically distinct subgroup in which co-alterations may be more prevalent than in unselected NSCLC cohorts. Second, the use of a comprehensive hybrid-capture sequencing platform (TSO500) with high coverage and systematic reporting of pathogenic and likely pathogenic variants may have increased the sensitivity for detecting RICTOR alterations compared to smaller or hotspot-based panels used in earlier studies.

In addition, the retrospective and multi-institutional nature of the dataset may have introduced an element of selection bias. In particular, cases with available broad-panel NGS data and adequate clinical annotation were preferentially included, and in some instances, patients with identified RICTOR alterations were specifically captured from participating centers to enable meaningful subgroup analyses. This approach, while allowing more detailed characterization of this rare molecular subset, may have contributed to an apparent enrichment of RICTOR mutations in the analyzed cohort. Furthermore, only pathogenic and likely pathogenic variants were included in the analysis, and variants of uncertain significance were excluded; however, differences in variant classification frameworks and reporting practices across datasets may also contribute to variability in reported mutation frequencies.

Taken together, these factors likely explain the higher observed frequency and should be considered when interpreting the generalizability of our findings. Therefore, our results are best viewed as hypothesis-generating, and external validation in larger, unselected, and prospectively collected datasets is warranted to better define the true prevalence and clinical impact of RICTOR mutations in EGFR-mutant NSCLC.

Beyond TKI resistance, we noted more frequent bone and pleural metastases at presentation in RICTOR-mutant patients. Although exploratory, this distribution is compatible with experimental links between mTORC2 signaling, epithelial–mesenchymal transition, enhanced motility, and niche-specific colonization [30, 31, 35]. These clinicobiologic observations warrant validation in larger datasets.

We did not observe a significant association between RICTOR status and PD-L1 expression in this cohort; however, emerging evidence suggests that mTORC2 influences tumor immunometabolism, potentially regulating checkpoint molecule expression and immune evasion [36, 37]. As immunotherapy assumes a greater role in EGFR-mutant NSCLC after TKI failure, the immunomodulatory consequences of RICTOR dysregulation merit prospective evaluation. Integrating immune profiling with genomic data may clarify whether RICTOR mutations also predict variable responsiveness to immune checkpoint blockade.

Interestingly, KRAS mutations (excluding G12C) were observed more frequently in the EGFR-mutant subgroup compared to EGFR-wild-type tumors. Although EGFR and KRAS are typically considered mutually exclusive oncogenic drivers, this pattern may reflect the inclusion of non-canonical KRAS alterations detected by comprehensive NGS profiling. These variants may represent subclonal events or co-occurring passenger alterations rather than dominant driver mutations, and therefore should be interpreted with caution. In addition, the relatively small sample size and retrospective design may have contributed to this unexpected distribution.

### Limitations

Despite the multi-center design, the retrospective nature of the study limits generalizability, and subgroup sample sizes constrain statistical power and increase the risk of model instability. We focused on mutation-level RICTOR alterations and did not evaluate copy-number changes; functional validation of individual variants was not performed. Treatment heterogeneity (including variable TKI generation and sequencing) reduced precision for genotype–treatment interaction estimates. Finally, correlative biomarkers (e.g., phospho-AKT, EMT markers) were not available to substantiate the proposed mechanisms. Although the median follow-up exceeded one year, providing sufficient survival maturity, the relatively small number of events within the EGFR-mutant subgroups warrants cautious interpretation of effect sizes, particularly the strong log-rank statistic observed.

## Conclusion

RICTOR mutations were associated with a high-risk subset of EGFR-mutant metastatic lung adenocarcinoma with substantially inferior survival under standard EGFR-directed therapy. Prospective, multicenter studies—integrating functional assays, immune profiling, and rational combination strategies targeting mTORC2/AKT—are needed to define the prognostic and predictive utility of RICTOR and to test therapeutic approaches tailored to RICTOR-altered disease.

## Supplementary Information

Below is the link to the electronic supplementary material.

Supplementary Material 1 (DOCX 18.3 KB)

## Data Availability

Additionally, as this study relies on institutional patient records, the data are not publicly available due to confidentiality agreements. Researchers interested in accessing the dataset may request it from the corresponding author, subject to institutional approval.
