# Supplementary material for: Prognostic significance of RICTOR mutations in EGFR-mutant metastatic lung adenocarcinoma: a retrospective cohort study
Source: Virchows Arch. 2026 May 4;489(1):79–90. doi: 10.1007/s00428-026-04559-2 (PMC13369718; doi:10.1007/s00428-026-04559-2)
Supplement: Supplementary file 1 — Supplementary Material 1 (DOCX 18.3 KB) [file 428_2026_4559_MOESM1_ESM.docx]

Supplementary Table S1. Detailed annotation of RICTOR variants identified in EGFR-mutant metastatic lung adenocarcinoma patients.

| **Patient ID** | **RICTOR Variant (cDNA)** | **Protein Change** | **Variant Type** | **VAF (%)** | **Pathogenicity** |
| --- | --- | --- | --- | --- | --- |
| **1** | c.4492G>A | p.D1498N | Missense | 24 | Likely Pathogenic |
| **2** | c.3895C>G | p.R1299G | Missense | 48 | Pathogenic |
| **3** | c.230C>T | p.A77V | Missense | 37 | Pathogenic |
| **4** | c.4897G>C | p.E1633Q | Missense | 33 | Pathogenic |
| **5** | c.4897G>C | p.E1633Q | Missense | 34 | Pathogenic |
| **6** | c.230C>T | p.A77V | Missense | 35 | Pathogenic |
| **7** | c.3332C>T | p.S1111L | Missense | 28 | Likely Pathogenic |
| **8** | c.3895C>G | p.R1299G | Missense | 38 | Pathogenic |
| **9** | c.4897G>C | p.E1633Q | Missense | 33 | Pathogenic |
| **10** | c.4492G>A | p.D1498N | Missense | 26 | Likely Pathogenic |
| **11** | c.3895C>G | p.R1299G | Missense | 48 | Pathogenic |
| **12** | c.4897G>C | p.E1633Q | Missense | 26 | Pathogenic |
| **13** | c.230C>T | p.A77V | Missense | 37 | Pathogenic |
| **14** | c.3895C>G | p.R1299G | Missense | 33 | Pathogenic |
| **15** | c.3895C>G | p.R1299G | Missense | 34 | Pathogenic |

This table summarizes the RICTOR variants detected by next-generation sequencing, including cDNA changes, corresponding protein alterations, variant type, variant allele frequency (VAF), and pathogenicity classification based on established databases and guidelines. Copy-number alterations were not included in this supplementary table because the present study focused on mutation-level RICTOR alterations, and amplification events were excluded from the primary analysis.

Variants classified as pathogenic or likely pathogenic were included in the analysis, while variants of uncertain significance were excluded. Several RICTOR variants were observed in more than one patient, reflecting recurrent alterations rather than unique patient-specific events.

Variant-level data were retrospectively extracted from original molecular reports, as these details were not systematically captured in the initial study database. Variant classification was based on curated databases (e.g., COSMIC, ClinVar, OncoKB).
